# Reliability of the ASA Physical Status Classification System in Predicting Surgical Morbidity: a Retrospective Analysis

**DOI:** 10.1007/s10916-021-01758-z

**Published:** 2021-07-22

**Authors:** Gen Li, Jeremy P. Walco, Dorothee A. Mueller, Jonathan P. Wanderer, Robert E. Freundlich

**Affiliations:** 1 Department of Anesthesiology, Vanderbilt University Medical Center, Nashville, TN USA; 2 Department of Anesthesiology, Department of Biomedical Informatics, Vanderbilt University Medical Center, Nashville, TN USA

**Keywords:** ASA physical status, Elixhauser comorbidities, Postoperative mortality, Comorbidity indices

## Abstract

The American Society of Anesthesiologists (ASA) Physical Status Classification System has been used to assess pre-anesthesia comorbid conditions for over 60 years. However, the ASA Physical Status Classification System has been criticized for its subjective nature. In this study, we aimed to assess the correlation between the ASA physical status assignment and more objective measures of overall illness. This is a single medical center, retrospective cohort study of adult patients who underwent surgery between November 2, 2017 and April 22, 2020. A multivariable ordinal logistic regression model was developed to examine the relationship between the ASA physical status and Elixhauser comorbidity groups. A secondary analysis was then conducted to evaluate the capability of the model to predict 30-day postoperative mortality. A total of 56,820 cases meeting inclusion criteria were analyzed. Twenty-seven Elixhauser comorbidities were independently associated with ASA physical status. Older patient (adjusted odds ratio, 1.39 [per 10 years of age]; 95% CI 1.37 to 1.41), male patient (adjusted odds ratio, 1.24; 95% CI 1.20 to 1.29), higher body weight (adjusted odds ratio, 1.08 [per 10 kg]; 95% CI 1.07 to 1.09), and ASA emergency status (adjusted odds ratio, 2.11; 95% CI 2.00 to 2.23) were also independently associated with higher ASA physical status assignments. Furthermore, the model derived from the primary analysis was a better predictor of 30-day mortality than the models including either single ASA physical status or comorbidity indices in isolation (p < 0.001). We found significant correlation between ASA physical status and 27 of the 31 Elixhauser comorbidities, as well other demographic characteristics. This demonstrates the reliability of ASA scoring and its potential ability to predict postoperative outcomes. Additionally, compared to ASA physical status and individual comorbidity indices, the derived model offered better predictive power in terms of short-term postoperative mortality.

## Introduction

The American Society of Anesthesiologists (ASA) physical status classification system was originally designed and implemented as a tool to summarize preoperative comorbidity status [1–4], and is now widely used in improving risk adjustment, determining reimbursement, and predicting perioperative risk and mortality [5–7], despite the explicit recommendation from the ASA House of Delegates that it not be used in isolation for risk stratification and mortality prediction [3]. However, due to the classification system’s largely subjective nature, there is inconsistent assignment of ASA physical status among clinicians [8, 9]. While there have been attempts to clarify exact definitions of the six classes, it is unclear how consistently they are applied in routine clinical practice [3, 10].

More recently, alternative major comorbidity indices have been created and validated to assess and summarize various patient conditions and their associated risks. The Charlson index was developed to predict 1-year mortality risk by assigning empirically derived scores to 19 clinically relevant comorbidities [11]. The Romano index, the most commonly used modification of Charlson index, groups the comorbidities on the basis of the International Classification of Diseases (ICD) diagnosis coding system (9^th^ & 10^th^ version) [12, 13]. A scoring system introduced by Elixhauser et al. predicts in-hospital mortality and length of stay [14] and, because it takes into account more comorbidities, may be superior to the Charlson/Romano index in predicting risk in certain surgical cohorts [15, 16]. Finally, van Walraven et al. condensed the Elixhauser comorbidities into a single numeric score, varying from -19 to 89, that reflected the association between overall comorbidity burden and death in hospital [17].

Despite the widespread use of the ASA physical status in routine clinical practice, it remains unclear how well it correlates with more objective models of risk prediction. Therefore, in this study we aim to pragmatically model the association between ASA physical status documented in routine clinical practice and specific comorbidities included in the aforementioned comorbidity indices. As a secondary analysis, we hypothesized that this model may outperform the ASA physical status and established comorbidity indices at predicting postoperative 30-day mortality.

## Methods

This manuscript adheres to the Strengthening the Reporting of Observational Studies in Epidemiology guidelines [18].

### Human subjects protection

This retrospective observational cohort study received approval from the Institutional Review Board (IRB) at Vanderbilt University Medical Center (IRB #190,231) with a waiver of the requirement for informed consent.

### Data collection

Patient data were extracted from the Vanderbilt University Medical Center Perioperative Data Warehouse (Nashville, TN) using Structured Query Language, from November 2, 2017 to April 22, 2020. All adult patients (≥ 18 years of age) who underwent anesthesia at Vanderbilt University Medical Center with an inpatient admission were eligible for inclusion in this retrospective study. For cases that met our inclusion criteria, relevant patient demographic data and clinical data were extracted, including patient age, sex, weight, body mass index, ASA physical status, ASA emergency status, the 31 Elixhauser comorbidities, van Walraven Score, Charlson-Deyo score, Romano score, and postoperative mortality (30-days, 60-days, and 90-days). Of note, at Vanderbilt University Medical Center, the ASA physical status was initially assigned by an anesthesia provider prior to surgery and confirmed (with or without modification) by the attending anesthesiologist. Meanwhile, the comorbidity diagnoses were assigned by coders at the conclusion of hospital admission. In addition, clinical documentation specialists also queried clinicians to add diagnoses and to provide further details. The individual Elixhauser comorbidities were identified using ICD diagnosis coding system (9^th^ & 10^th^ version), and coding algorithms were used to calculate the various comorbidity indices (van Walraven score, Charlson-Deyo score, and Romano score) following extraction of those diagnoses from the medical records [11, 13, 17]. Cases with ASA physical status category 6, denoting a declared brain-dead organ donor, were excluded. For the cases with missing data or conspicuous errors (e.g., body mass index < 10 kg/m^2^) in terms of demographic datapoints, data were retrieved from a separate encounter of the same patient within two years, if applicable. Clinical missing data that were considered missing at random were imputed with median values.

### Statistical analysis

Demographic, clinical, and procedural variables were used to characterize the study population with means and standard deviations (SDs) for parametric variables, with medians and interquartile ranges for nonparametric variables, and with percentages for categorical variables.

The primary analysis was conducted using multivariable ordinal logistic regression. The ordinal ASA physical status was treated as the dependent outcome variable. Thirty-one Elixhauser comorbidities were treated as independent variables in the regression model and other significant variables, including age, gender, weight, body mass index, type of surgery, and duration of surgery, were controlled as covariates in the regression. A Least Absolute Shrinkage and Selection Operator approach was applied to identify covariates for inclusion in the regression model. To ensure the reliability and robustness of model performance, a five-fold cross validation procedure was conducted for developing the best fitting model [19], and the Least Absolute Shrinkage and Selection Operator approach selected predictors were applied to an ordinal logistic regression to fit the final model. The quality of the fit was evaluated by the area under the curve (AUC) of the Receiver Operating Characteristic curve, and the variance inflation factor was computed to detect potential collinearity, by assessing the variance change of an estimated regression coefficient [20]. The associations were then summarized and reported using the ordinal odds ratios with 95% confidence intervals (CIs), and tested using the Wald multiple degree of freedom Chi-squared test.

The ordinal odds ratio demonstrates the relative odds of the incident of the outcome of interest, given exposure to the other covariates for the ordinal logistic regression model, and should be interpreted as follows: The ordinal odds are the probability that Y ≥ y divided by one minus itself $\left(\frac{P}{1-P}\right)$, in which Y denotes the ordinal ASA physical status and y (e.g., 1, 2, 3, 4, or 5) denotes one of its levels [21–23]. Therefore, the odds ratio should be interpreted as the fold-change in the odds of the next higher ASA physical status associated with a change in the corresponding covariate after controlling for all the other covariates.

Next, to determine the relationship between the ASA physical status and composite comorbidity indices, a prespecified sensitivity analysis was then conducted using van Walraven score, Charlson-Deyo score, and Romano score as independent variables in the regression model.

In addition, secondary analyses were performed to assess the ability of the composite regression model that derived from primary analysis to predict postoperative 30-day mortality. As a measure of discrimination, the c-statistic was calculated to evaluate the predictive capability, and the calibration plot was generated to examine whether the model was correctly specified. We also computed c-statistic and its corresponding 95% CI for the univariate model of ASA physical status and the model that derived from sensitivity analysis and compared these values across three models.

A two-sided hypothesis testing with p-value of less than 0.05 was deemed to indicate statistical significance. Statistical analyses were performed in SAS 9.4 (SAS Institute Inc., Cary, NC, USA).

## Results

Between November 2017 and April 2020, there were 56,932 adult perioperative admissions identified in the Vanderbilt University Medical Center Perioperative Data Warehouse database. From these, 112 ASA physical status 6 cases were excluded from analysis. Twenty-three (0.04%) patients with no registered ASA physical status and three (0.005%) patients with no registered ASA emergency status were imputed with median values. As an example, in the cohort, we found that a few cardiac surgery cases for decompensated heart failure and implementation of mechanical circulatory support were assigned an ASA physical status 1 or 2. While these assignments seemed to be inappropriate, they were retained in the cohort to preserve data integrity. Thus, a total of 56,820 surgical cases that met our inclusion criteria were analyzed in this study. Patients had a mean age of 53.1 years (SD = 18.0) and 51.3% were male. The ASA score was greater than 2 in 74.6% of patients, and 1,961 (3.5%) patients died within 30 days after surgery. Detailed patient characteristics are listed in Table 1.

**Table 1:** Demographic Characteristics of the Study Sample

Variables	Cases (N = 56,820)
**Age** in Years, mean (SD)	53.1 (18.0)
**Body Mass Index** in kg/m^2^, median (Interquartile Range)	28.5 (24.4–33.8)
**Weight** in kg, median (Interquartile Range)	83.9 (70.0–99.8)
**Gender** (%)	
Female	27,673 (48.7%)
**ASA Physical Status** (%)	
1	1,097 (1.9%)
2	13,396 (23.6%)
3	30,925 (54.4%)
4	10,979 (19.3%)
5	423 (0.8%)
**ASA Emergency** (%)	6,813 (12.0%)
**van Walraven Index** (%)	
< 0	10,302 (18.1%)
0	14,163 (24.9%)
> 0	32,355 (57.0%)
**Charlson Index** (%)	
< 4	41,256 (72.6%)
4–6	10,583 (18.6%)
≥ 7	4,981 (8.8%)
**Romano Index** (%)	
< 4	36,494 (64.2%)
4–6	11,365 (20.0%)
≥ 7	8,961 (15.8%)
**Mortality** (%)	
30 days	1,961 (3.5%)
60 days	2,471 (4.4%)
90 days	2,784 (4.9%)

From the results of multivariable ordinal logistic regression model, we found that 27 of the 31 Elixhauser comorbidities were associated with ASA physical status (Table 2). Adjusted odds ratios ranged from 0.78 (95% CI: 0.69 to 0.88) for blood loss anemia to 2.57 (95% CI: 2.42 to 2.73) for congestive heart failure. Five of the 27 comorbidities were independently associated with a decreased likelihood of a patient receiving higher assignment of ASA physical status, while the remaining 22 comorbidities were independently associated with an increased likelihood of the primary outcome measure. Twenty-five of the comorbidities were selected by Least Absolute Shrinkage and Selection Operator approach and retained in the final model, along with other significant factors, including older age (adjusted odds ratio, 1.39 [per 10 years of age]; 95% CI 1.37 to 1.41; p < 0.001), male gender (adjusted odds ratio, 1.24; 95% CI 1.20 to 1.29; p < 0.001), higher body weight (adjusted odds ratio, 1.08 [per 10 kg]; 95% CI 1.07 to 1.09; p < 0.001), and the ASA emergency status (adjusted odds ratio, 2.11; 95% CI 2.00 to 2.23; p < 0.001).

**Table 2:** Prevalence of Elixhauser Comorbidities and Their Associations with ASA Physical Status

Elixhauser Group	Cases (%)	Adjusted OR (95% CI)*	P-Value
Congestive Heart Failure	9,483 (16.7%)	2.57 (2.42, 2.73)	< .001
Cardiac Arrhythmias	17,466 (30.7%)	1.42 (1.36, 1.48)	< .001
Valvular Disease	4,889 (8.6%)	1.90 (1.78, 2.04)	< .001
Pulmonary Circulation Disorders	4,366 (7.7%)	1.49 (1.39, 1.60)	< .001
Peripheral Vascular Disorders	6,224 (11.0%)	1.27 (1.19, 1.34)	< .001
Hypertension, Uncomplicated	25,979 (45.7%)	1.21 (1.17, 1.26)	< .001
Hypertension, Complicated	6,300 (11.1%)	0.87 (0.80, 0.94)	0.001
Paralysis	1,948 (3.4%)	1.85 (1.68, 2.04)	< .001
Neurodegenerative Disorders	3,580 (6.3%)	1.38 (1.29, 1.48)	< .001
Chronic Pulmonary Disease	11,790 (20.7%)	1.23 (1.17, 1.28)	< .001
Diabetes, Uncomplicated	8,545 (15.0%)	0.97 (0.92, 1.02)	0.219
Diabetes, Complicated	10,020 (17.6%)	1.30 (1.23, 1.37)	< .001
Hypothyroidism	7,674 (13.5%)	1.01 (0.96, 1.06)	0.821
Renal Failure	9,676 (17.0%)	1.70 (1.58, 1.84)	< .001
Liver Disease	6,511 (11.4%)	1.48 (1.39, 1.57)	< .001
Peptic Ulcer	1,497 (2.6%)	0.99 (0.88, 1.10)	0.770
AIDS/HIV	484 (0.9%)	1.50 (1.24, 1.81)	< .001
Lymphoma	648 (1.1%)	0.82 (0.70, 0.97)	0.017
Metastatic Cancer	4,676 (8.2%)	1.10 (1.01, 1.19)	0.022
Solid Tumor	9,359 (16.5%)	0.82 (0.77, 0.87)	< .001
Rheumatoid Arthritis	2,391 (4.2%)	1.12 (1.03, 1.22)	0.008
Coagulopathy	8,396 (14.8%)	1.87 (1.77, 1.98)	< .001
Obesity	12,205 (21.4%)	1.21 (1.15, 1.27)	< .001
Weight Loss	9,949 (17.5%)	1.46 (1.38, 1.53)	< .001
Fluid & Electrolyte Disorders	19,589 (34.4%)	2.13 (2.03, 2.23)	< .001
Blood Loss Anemia	1,229 (2.2%)	0.78 (0.69, 0.88)	< .001
Deficiency Anemia	4,112 (7.2%)	0.87 (0.81, 0.93)	< .001
Alcohol Abuse	2,319 (4.1%)	1.11 (1.01, 1.21)	0.027
Drug Abuse	3,552 (6.3%)	1.21 (1.12, 1.30)	< .001
Psychoses	523 (0.9%)	0.98 (0.82, 1.17)	0.828
Depression	10,677 (18.8%)	1.05 (1.00, 1.10)	0.046

^*^The adjusted odds ratio provides the association of the ASA physical status change (an increase in ASA score by 1 point) and the Elixhauser group after adjusting for all other groups and covariates in the multivariable ordinal logistic regression

**Fig. 1:**
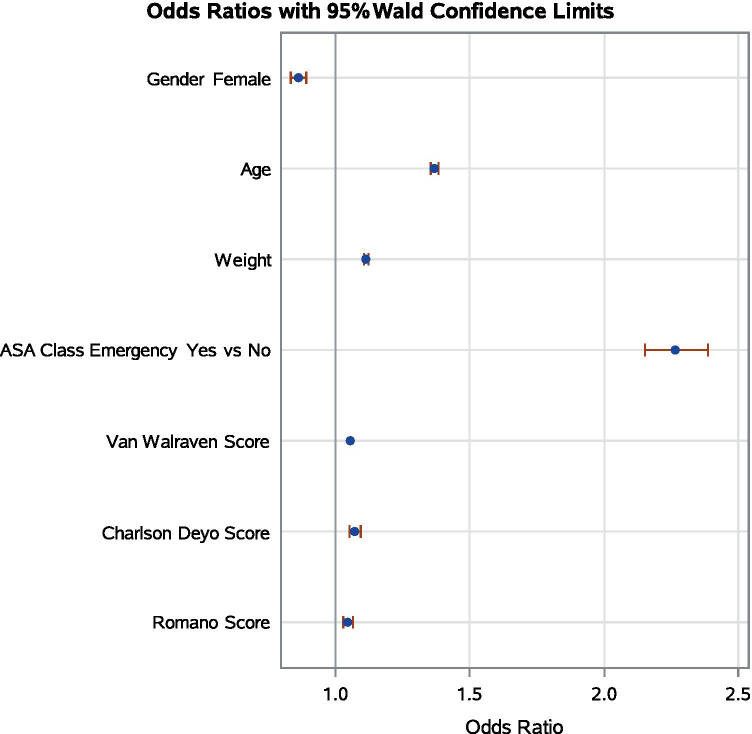
Visualization of the sensitivity analysis results that derived from multivariable ordinal logistic regression model. The odds ratio estimates and their corresponding 95% Wald confidence intervals demonstrate the odds of the next higher ASA physical status associated with the change in the corresponding covariates. For continuous variables, the odds ratios correspond to a unit increase in the risk factors

We further confirmed the associations between the ASA physical status and composite comorbidity indices from the sensitivity analysis. The actual ranges of van Walraven scores, Charlson-Deyo scores and Romano scores in our study cohort were—14 to + 67, 0 to 19, and 0 to 22, respectively. All three comorbidity indices were independently associated with the ASA physical status with adjusted odds ratio of 1.055 per one unit increase (95% CI 1.053 to 1.058; p < 0.001) for van Walraven scores, 1.07 per one unit increase (95% CI 1.05 to 1.10; p < 0.001) for Charlson-Deyo scores, and 1.05 per one unit increase (95% CI 1.03 to 1.07; p < 0.001) for Romano scores (Fig. 1).

**Fig. 2:**
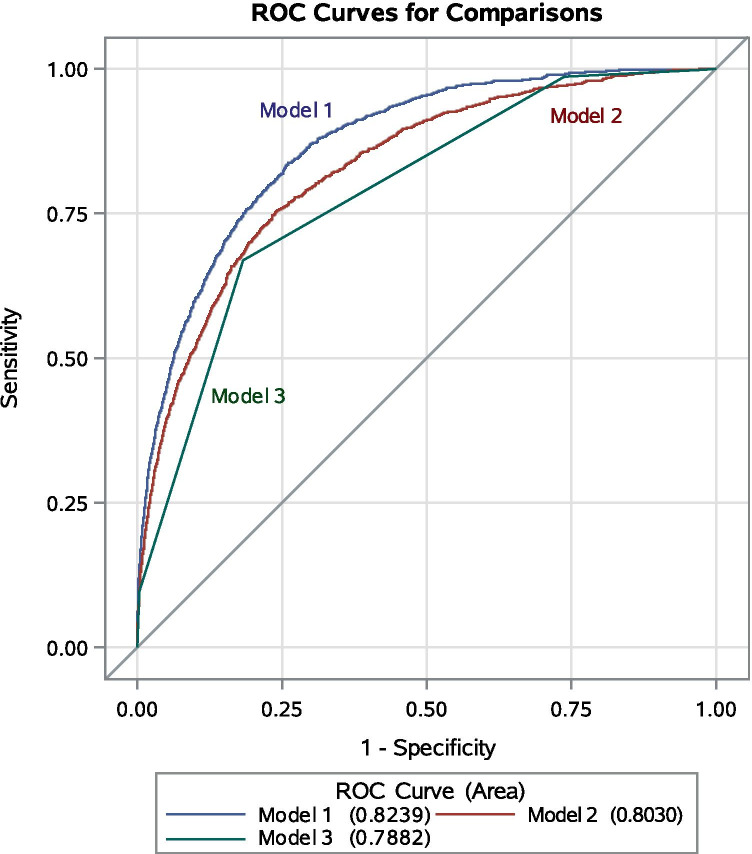
Visualization of the Receiver Operating Characteristic (ROC) curves comparison in terms of the predictive capability of models for postoperative 30-day mortality. Model 1 consists of the covariates that derived from primary analysis, model 2 consists of the covariates that derived from sensitivity analysis, and model 3 consists of the ASA physical status

Compared with the univariate model (Model 3) using ASA physical status as a predictor for 30-day mortality after surgery (c = 0.79; 95% CI 0.78 to 0.80), the c-statistic for the model (Model 1) that derived from primary analysis was 0.82 (95% CI 0.81 to 0.83), and for the composite comorbidity indices model (Model 2) that that derived from sensitivity analysis was 0.80 (95% CI 0.79 to 0.81) (Fig. 2).

## Discussion

Given the widespread clinical, billing, and research use of the ASA Physical Status Classification System for risk stratification, the assessment of correlation between ASA physical status assignment and a patient’s comorbidity burden is very important. Our study benefits from its inclusion of almost 57,000 surgical patients, a large portion of which have significant comorbidities, as well its robust statistical analysis. We demonstrate a clear association between the ASA physical status and more objective patient demographics and comorbidity measures, as a total of 27/31 (87.1%) Elixhauser comorbidities were independently associated with the ASA physical status, except uncomplicated diabetes, hypothyroidism, peptic ulcer and psychoses.

Among those 27 Elixhauser comorbidities, five (blood loss anemia, solid tumor, lymphoma, deficiency anemia, and complicated hypertension) were actually associated with a lower ASA physical status. This may reflect bias on the part of anesthesia providers, who may be more concerned about neurologic, pulmonary, and cardiovascular comorbidities, and unduly weight their importance. Another possible explanation for this apparent contradiction could be a coding bias proposed by Elixhauser: namely, the severity of a patient’s overall illness inversely affects the likelihood that some certain conditions are coded [14]. Bias may have been similarly influential in our finding that older age, higher body weight, male gender, and ASA emergency status were also independently associated with higher ASA physical status assignments.

Whereas the other indices are used in both surgical and medical patients, the ASA classification is assigned in perioperatively setting, and becomes a readily available assessment for patient preoperative risk stratification. Although it was not developed for predicting postoperative mortality and risk, numerous studies have been performed to determine the association between the ASA physical status and postoperative outcomes. A multi-institutional cohort study of more than two million cases from the American College of Surgeon National Surgical Quality Improvement Program database demonstrated an association between ASA physical status and postoperative medical complications, including mortalities [6]. Boorjian et al. indicated that, compared to van Walraven and Charlson comorbidity indices, the ASA physical status assignment was superior in predicting 90-day mortality following radical cystectomy [24]. Similarly, a retrospective cohort study of 226 patients who underwent spinal surgery showed that an increasing ASA physical status score was associated with both a higher occurrence of major complications and increased direct cost [25]. Our study adds significant credibility to these findings and provides a more comprehensive picture of the association between the ASA physical status, Elixhauser comorbidities, and other commonly used comorbidity indices, regardless of the type of surgery. We demonstrated that when assigning ASA physical status to patients, providers captured similar elements of patients’ preoperative comorbidity burdens as those that were more objectively measured by the van Walraven, Charlson-Deyo, and Romano indices. Thus, the ASA Physical Status Classification System appears to effectively enable providers to summarize and quantify comorbidity statuses, despite its subjective nature and can be used to reliably assess perioperative risk.

While we observed an association between the ASA physical status and twenty-seven Elixhauser comorbidities in this study, the ASA physical status may need more modifications to improve correct assignment by increasing its reliability and decreasing subjectivity [26], especially when scored by non-anesthesia clinicians [10]. Recently, a cross-sectional study of 229 clinicians from various specialties described disagreement between ASA score assignments by surgeons and anesthesiologists, as well as a higher degree of variability and lower scores by internal medicine providers and procedural staff than by anesthesia providers [27]. Anecdotally, although an ASA physical status of 1 or 2 is often cited by guidelines as criteria that do not require as invasive of monitoring and present lower risk [10], in the conduct of reviewing data for this study, we found multiple instances of grossly inappropriate ASA physical status. In practice, these cases are likely to be clerical errors or differing views on how to appropriately classify an otherwise healthy patient with an acute illness. It is likely that more objective data provided at the time of ASA physical status assignment could potentially improve appropriate and consistent assignment of ASA physical status.

There are several limitations to this study. While the ASA Physical Status Classification System has been a useful risk stratification tool at our medical center, further modifications and more institutional-specific examples would be necessary to supplement the ASA-approved examples for providers to improve the accuracy of assignment. Furthermore, since the c-statistic is insensitive to inclusion of significant factors in a model, it has been critiqued for its ability to assess a model’s predictive capability [28]. Therefore, we also assessed the calibrations of different models. However, a multicenter analysis may be warranted for the model assessment, and further examinations of the composite model in surgery-specific subpopulations would also necessary. Given the outstanding performance of our multivariable composite model, the issue of overfitting should not be ignored, especially when evaluating the risk of rare events (i.e. postoperative short-term mortality). Another challenge with this model is that it requires data that is available only at the end of the hospital admission, whereas the ASA physical status is assigned preoperatively. There is a limitation to being able to identify which comorbidities were present the day of surgery, as they might have only been charted later throughout the hospital stay, while nearly all comorbidities are chronic diseases. Moreover, the availability of all comorbidities in the chart is reliant on the postoperative team to chart these even if they are not in relation to their primary diagnoses. However, despite its limitations, the model proved promising in predicting risk of short-term postoperative mortality. Finally, the conclusions of our study are limited by its retrospective nature [29].

## Conclusion

In summary, we provide evidence of a correlation between the ASA physical status and 27 of the 31 Elixhauser comorbidities, lending significant credence to the scoring system’s reliability and its capacity to accurately predict perioperative outcomes. Additionally, our composite model of various elements of the Elixhauser comorbidity index and other patient characteristics better predicted short-term postoperative mortality than both the ASA physical status and other widely used comorbidity indices. This opens the door for further studies aimed at refining risk assessment of surgical patients.
